# Hindsight/RREB-1 functions in both the specification and differentiation of stem cells in the adult midgut of *Drosophila*

**DOI:** 10.1242/bio.015636

**Published:** 2015-12-10

**Authors:** Brittany L. Baechler, Cameron McKnight, Porsha C. Pruchnicki, Nicole A. Biro, Bruce H. Reed

**Affiliations:** Department of Biology, University of Waterloo, 200 University Avenue West, Waterloo, Ontario N2L 3G1, Canada

**Keywords:** Hindsight/RREB-1, Intestinal stem cells, Enterocyte, Differentiation

## Abstract

The adult *Drosophila* midgut is established during the larval/pupal transition from undifferentiated cells known as adult midgut precursors (AMPs). Four fundamental cell types are found in the adult midgut epithelium: undifferentiated intestinal stem cells (ISCs) and their committed daughter cells, enteroblasts (EBs), plus enterocytes (ECs) and enteroendocrine cells (EEs). Using the *Drosophila* posterior midgut as a model, we have studied the function of the transcription factor Hindsight (Hnt)/RREB-1 and its relationship to the Notch and Egfr signaling pathways. We show that *hnt* is required for EC differentiation in the context of ISC-to-EC differentiation, but not in the context of AMP-to-EC differentiation. In addition, we show that *hnt* is required for the establishment of viable or functional ISCs. Overall, our studies introduce *hnt* as a key factor in the regulation of both the developing and the mature adult midgut. We suggest that the nature of these contextual differences can be explained through the interaction of *hnt* with multiple signaling pathways.

## Abstract (precis)

**Summary:** Hindsight, the *Drosophila* homolog of RREB-1, can promote and is required for enterocyte differentiation specifically in the adult midgut. Enterocyte differentiation during the larval/pupal transition does not require Hindsight.

## INTRODUCTION

Until relatively recently, it was a commonly held view that differentiated somatic tissues of *Drosophila* adults are static and non-proliferative. Lineage tracing techniques, however, have demonstrated the existence of stem cells within several tissues including intestinal stem cells (ISCs) within the posterior midgut (Micchelli and Perrimon, 2006; Ohlstein and Spradling, 2006; Singh et al., 2007). ISC regulation is remarkably conserved between mammals and *Drosophila* (Casali and Batlle, 2009; Hartenstein et al., 2010; Wang and Hou, 2010). Studies using *Drosophila* can, therefore, contribute to our understanding of stem cell biology and associated human diseases.

The adult *Drosophila* midgut arises from mitotically active cells of the embryonic endoderm (Hartenstein et al., 2010). These cells are marked by the expression of *escargot* (*esg*), which encodes a C_2_H_2_-type Zinc finger protein that is required for the maintenance of diploidy in several tissues (Hayashi et al., 1993; Korzelius et al., 2014). *esg* expression is maintained in cells that will become the adult midgut precursors (AMPs) (Takashima et al., 2011). During late stages of embryogenesis, the AMPs, which remain undifferentiated, migrate through the newly formed midgut to become situated along the basal surface of the epithelium (Jiang and Edgar, 2009; Takashima et al., 2011). During the third larval instar, AMPs are found as clusters in which 1-3 AMPs differentiate in a Notch-dependent manner to form a specialized cell type, the peripheral cell (PC), which enwraps the remaining eight or more undifferentiated AMPs at this stage (Mathur et al., 2010; Takashima et al., 2011).

During the larval/pupal transition, the differentiated larval enterocytes (ECs) and enteroendocrine cells (EEs) are eliminated by autophagic cell death (Denton et al., 2009). Most AMPs differentiate to form the adult ECs, while some remaining AMPs become ISCs (Takashima et al., 2011). The mechanism of AMP-to-ISC specification is not understood. AMP-to-EC differentiation, however, can occur through a pathway parallel to Notch signaling (see below) that requires ecdysone signaling and the transcriptional regulator Broad. Ecdysone signaling and Broad are also implicated in the differentiation of fully functional ISCs from AMPs (Zeng and Hou, 2012).

The *hindsight* (*hnt*) gene encodes a nuclear protein containing 14 C_2_H_2_-type Zinc fingers. The expression of *hnt* during development is complex and dynamic (Yip et al., 1997). In general, despite a wealth of information, a detailed understanding of how Hnt functions to regulate cellular and developmental processes has remained elusive (Oliva et al., 2015; Pickup et al., 2009; Sun and Deng, 2007; Wilk et al., 2000).

Ras responsive element binding protein-1 (RREB-1), the mammalian homologue of Hnt, can act as either a transcriptional repressor or activator, depending on the context and target gene (Liu et al., 2009; Thiagalingam et al., 1996). Recent studies suggest that Hnt and RREB-1 are functionally conserved (Ming et al., 2013). In humans, RREB-1 has been linked to pancreatic, thyroid, and colorectal cancer (Kent et al., 2013; Zhang et al., 2003).

In *Drosophila*, ISCs are marked by the expression of *esg* and can divide either symmetrically or asymmetrically (de Navascues et al., 2012). Asymmetric divisions typically produce one daughter cell that retains the ISC identity and a second committed daughter cell known as an enteroblast (EB) (Micchelli and Perrimon, 2006; Ohlstein and Spradling, 2006). EBs also express *esg* and differentiate without dividing to become either ECs or EEs; the former undergo endoreduplication and become polyploid, while the latter remain diploid (Strand and Micchelli, 2013; Zeng et al., 2013a). ISCs express the Notch ligand Delta, and EBs that receive a higher level of Notch activation differentiate as ECs, whereas EBs receiving a lower level of Notch activation differentiate as EEs (Ohlstein and Spradling, 2007). Reduced Notch signaling results in uncontrolled ISC division, decreased EC differentiation, and an increased number of EE-like cells (Micchelli and Perrimon, 2006; Ohlstein and Spradling, 2006, 2007), while overexpression of activated Notch promotes EC differentiation (Micchelli and Perrimon, 2006; Ohlstein and Spradling, 2007). Interestingly, *hnt* has been identified as a Notch-responsive gene and its expression has been shown to be Notch-dependent in some contexts (Krejci et al., 2009; Sun and Deng, 2007; Terriente-Felix et al., 2013).

The Egfr/Ras/MAPK signaling pathway (hereafter the Egfr pathway) is required for ISC proliferation (Biteau and Jasper, 2011; Buchon et al., 2010; Jiang and Edgar, 2009). Over-activation of Egfr signaling results in increased ISC proliferation and midgut hyperplasia (Biteau and Jasper, 2011; Buchon et al., 2010; Jiang and Edgar, 2009). While Egfr signaling promotes ISC proliferation, it does not influence subsequent differentiation events (Biteau and Jasper, 2011).

An additional pathway regulating ISC proliferation is the JAK/STAT pathway. Activation of this pathway in ISCs leads to increased ISC proliferation while reduced JAK/STAT signaling leads to an accumulation of EB-like cells, suggesting that this pathway is required for the competence of EB cells to undergo EC or EE differentiation (Beebe et al., 2010).

As summarized in several recent reviews, the list of signaling pathways and genes regulating midgut development, homeostasis, and regeneration has become extensive (Buchon et al., 2014; Kux and Pitsouli, 2014; Naszai et al., 2015; Tipping and Perrimon, 2014; Zeng et al., 2013a). Several studies have recently expanded our understanding of both EC and EE differentiation. Regarding the former, the expression of *esg* has been found to suppress EC differentiation through the repression of EC-specific genes such as *Pdm1* (Korzelius et al., 2014). Additionally, BMP signaling (Dpp/Gbb) as well as the chromatin remodeling proteins Brahma and Osa have been shown to be required for proper EC differentiation (Jin et al., 2013; Zeng et al., 2013b; Zhou et al., 2015). EE differentiation is promoted by the proneural genes *asense* and *scute*, with the transcriptional regulation of *asense* being Osa-dependent (Bardin et al., 2010; Zeng et al., 2013b). More recently, robo/slit signaling has been found to regulate a negative feedback mechanism that limits EE regeneration (Biteau and Jasper, 2014).

This study represents the first detailed analysis of the expression and function of *hnt* in the adult midgut. We report that ISCs/EBs express *hnt* and that this expression is increased in differentiated ECs and is absent from EEs. We find *hnt* expression in ISCs/EBs to be independent of Notch signaling and Egfr-dependent. In addition, we show that *hnt* overexpression induced in ISCs/EBs results in EC differentiation and we conclude that *hnt* can promote, but is not sufficient, for EC differentiation. Qualitative and quantitative mosaic analysis of loss-of-function alleles demonstrates a requirement for *hnt* in ISC-to-EC differentiation, but not in AMP-to-EC differentiation. An additional and novel finding of our study is the requirement for *hnt* in the establishment of viable or functional ISCs. Overall, our work ascertains that the transcriptional regulator Hnt/RREB-1 is an important component of the developing and homeostatic adult midgut where it functions in the both the specification and subsequent differentiation of ISCs.

## RESULTS

### *hnt* is expressed in the adult intestinal epithelium

**Fig. 1.**
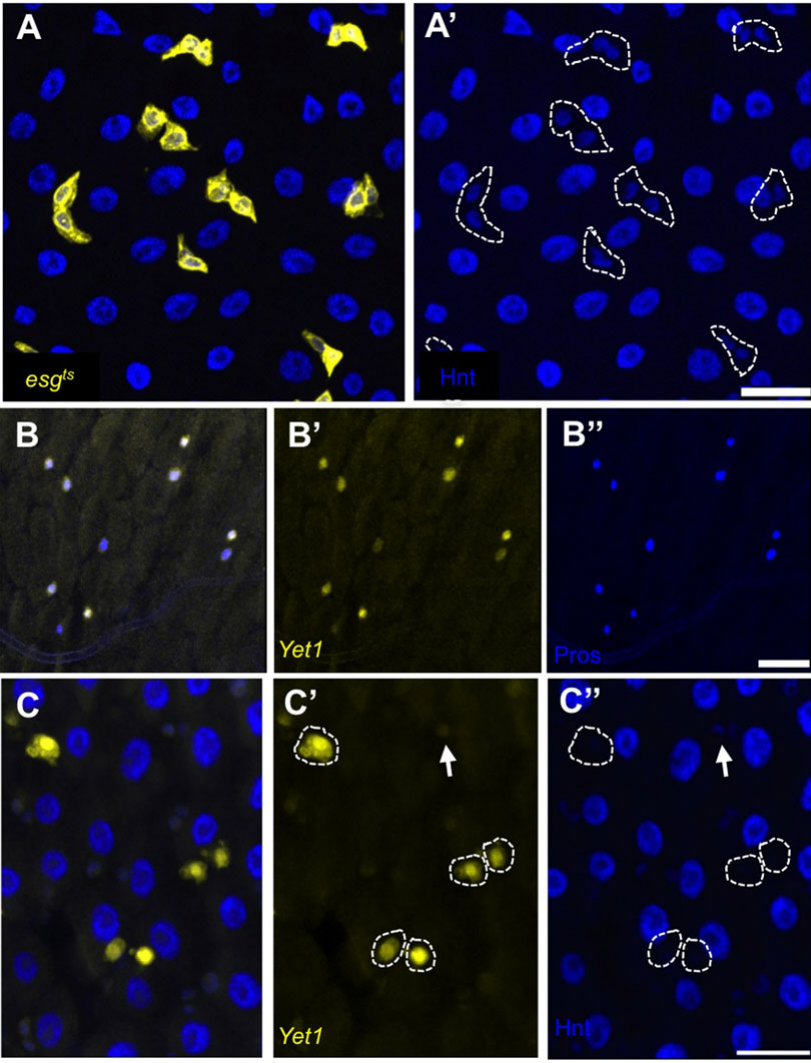
***hnt* is differentially expressed in the adult ISC lineage and is not expressed in mature EEs.** (A,A′) Anti-Hnt immunostaining (blue) of adult midgut in which ISCs/EBs are marked by GFP expression (yellow) using the *esg^ts^* technique (see Materials and Methods) showing *hnt* expression in ISCs/EBs (outlines in A′) and ECs (large GFP-negative cells). Hnt levels appear higher in ECs compared to ISCs/EBs. (B-B″) Anti-Pros immunostaining (blue) of the GFP enhancer trap line *Yet1* demonstrates co-localization of Pros and GFP (yellow). (C-C″) Anti-Hnt immunostaining (blue) of *Yet1* confirms that strong GFP-positive (yellow) EEs are Hnt-negative (outlines) while weak GFP-positive cells, likely immature EEs, show very low Hnt signal (arrows). Scale bars: 20 µm.

The Notch signaling pathway is required for the normal maintenance and regeneration of the adult midgut (Micchelli and Perrimon, 2006; Ohlstein and Spradling, 2006). The gene *hnt* has been identified as a target of the Notch signaling pathway, but in contexts other than that of the adult midgut (Krejci et al., 2009; Sun and Deng, 2007; Terriente-Felix et al., 2013). To investigate the possible functions of Hnt in the adult midgut, and to determine if *hnt* is a target of Notch signaling in this context, we performed anti-Hnt immunostaining of adult midguts. Immunostaining of *esg^ts^* midguts following shift to permissive conditions (see Materials and Methods) allowed unambiguous identification of ISCs/EBs as Hnt-positive. Large, polyploid, GFP-negative cells corresponding to ECs were also Hnt-positive (Fig. 1A). In general, the intensity of anti-Hnt signal in the *esg^ts^-*marked ISCs/EBs was less than the signal observed in ECs. In addition, small GFP-negative cells were sometimes observed to be weakly Hnt-positive (data not shown), possibly representing EEs. Unfortunately, anti-Hnt and the EE marker anti-Prospero (Pros) are both mouse monoclonal antibodies, which precluded a double immunolabeling experiment. To circumvent this problem we used a GFP enhancer trap line, *Yet1*, which we here report as a new EE marker. Anti-Pros immunostaining of *Yet1* adult midguts confirmed that *Yet1* expression and Pros co-localize (Fig. 1B). Anti-Armadillo (Arm) immunostaining also confirmed that *Yet1*-expressing cells are single small cells associated with reduced Arm, consistent with EE morphology (Fig. S1). Anti-Hnt immunostaining of the *Yet1* line subsequently established that strong GFP-positive cells are Hnt-negative (Fig. 1C), while weakly GFP-positive cells are sometimes weakly Hnt-positive (arrows, Fig. 1C). We conclude that strong *Yet1* expression marks differentiated EEs and that this cell type does not express *hnt*. These observations also suggest that *hnt* is down-regulated in EBs that are specified to become EEs.

### ISC expression of *hnt* is not dependent on Notch signaling but is dependent on Egfr signaling

**Fig. 2.**
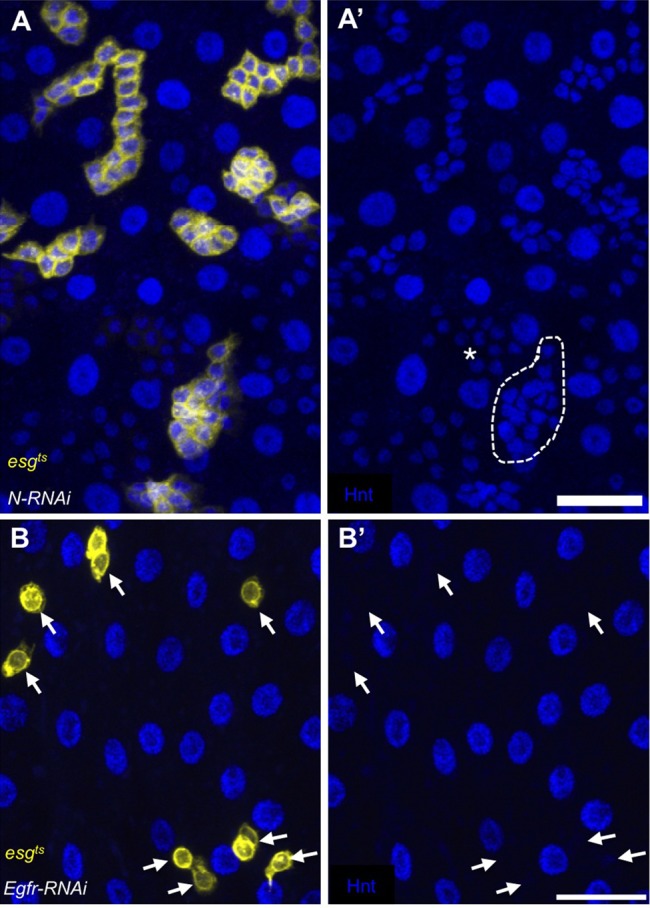
***hnt* ISC/EB expression is independent of Notch signaling, but is dependent on Egfr signaling.** (A-A′) Anti-Hnt (blue) immunostained midgut depleted of Notch by RNAi expression using the *esg^ts^* technique showing tumor-like clusters of GFP-positive (yellow; A′, outline) and GFP-negative cells (A′, asterisk). Both types of overproliferating cells are Hnt-positive. (B,B′) RNAi mediated depletion of Egfr in ISCs/EBs results in a reduced, Hnt-negative ISC/EB population (arrows). Scale bars: 20 µm.

To address the expression of *hnt* in the context of reduced Notch signaling, anti-Hnt immunostaining was performed on midguts in which Notch was depleted by expression of *UAS-Notch-RNAi* using the *esg^ts^* technique. Consistent with previous findings (Micchelli and Perrimon, 2006; Ohlstein and Spradling, 2006), we found that midguts with reduced Notch signaling display an overproliferation of two cell types which we observed as small GFP-positive, Hnt-positive cells, as well as small GFP-negative, Hnt-positive cells (Fig. 2A). Our above finding that Hnt is not expressed in differentiated EEs supports the view that the small GFP-negative, Hnt-positive cells represent an intermediate cell type that is neither a fully differentiated EE nor an ISC. In the GFP-positive over-proliferating ISC-like cells, however, we found no indication of reduced *hnt* expression. Taken together, these observations support the interpretation that ISCs do not express *hnt* in a Notch-dependent manner.

The mammalian homologue of *hnt* is *RREB-1*, and several lines of evidence suggest that RREB-1 functions in the regulation of Egfr signaling (Kent et al., 2013, 2014; Thiagalingam et al., 1996). Moreover, *Egfr* and *hnt* mutants share a number of phenotypes, including premature degeneration and death of the extra-embryonic tissue known as the amnioserosa (Frank and Rushlow, 1996; Shen et al., 2013). For these reasons we examined the expression of *hnt* in the context of reduced Egfr signaling. Midguts depleted of Egfr by expression of *UAS-Egfr-RNAi* were sparsely populated by GFP-positive cells (ISCs/EBs), and these were uniformly Hnt-negative (Fig. 2B). Thus, while we found no dependence on Notch signaling, we find that *hnt* expression in ISCs/EBs is dependent on Egfr signaling.

### Increased *hnt* expression forces ISC to EC differentiation

**Fig. 3.**
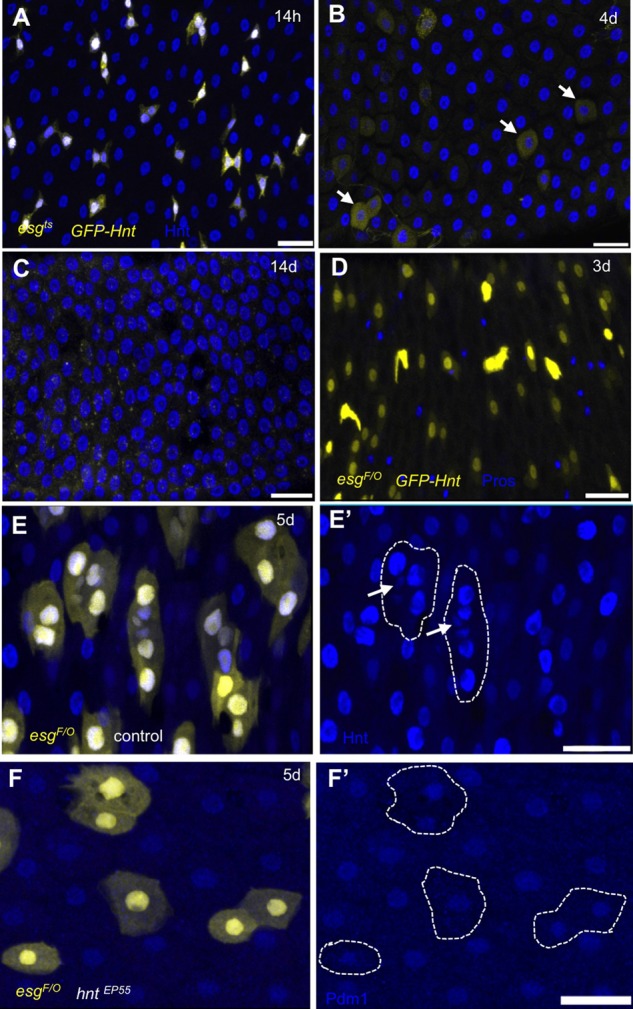
***hnt* overexpression forces ISC to EC differentiation.** (A-C) anti-Hnt (blue) immunostained adult midguts expressing GFP-Hnt and GFP (yellow) using the *esg^ts^* technique are shown at 14 h, 4 days, and 14 days following the shift to GAL4 permissive conditions. At 4 days post shift only weakly GFP-positive large cells, characteristic of differentiated ECs, are observed (B, arrows). No GFP-positive cells remain outside of the gastric region 14 days post shift (C). (D) Anti-Pros (blue) immunostained midgut expressing GFP-Hnt and GFP (yellow) using the *esg^F/O^* technique (see Materials and Methods) showing no co-localization of GFP and Pros. (E) Anti-Hnt (blue) immunostained control 5 days post shift showing clusters of GFP-positive cells. Control ISC lineages consist of several large cells (E′, outlines) and small cells (E′, arrows), consistent with ECs and ISCs/EBs, respectively. (F) Anti-Pdm1 (blue) immunostained adult midgut overexpressing *hnt^EP55^* showing GFP and Pdm1-positive cells 5 days post shift. ISC lineages overexpressing *hnt* consist of only one or two large Pdm1-positive cells (F′, outlines). Scale bars: 20 µm.

Our initial observations suggested that *hnt* expression may increase in association with EC specification, and decrease during EE differentiation. We were initially interested in determining if *hnt* overexpression in ISCs/EBs (using the *esg^ts^* technique) could bias specification towards the EC fate. What we found, however, was a striking loss of all ISCs/EBs. As early as 14 h post shift, ISCs/EBs with high levels of Hnt and a slight increase in size were observed (Fig. 3A). At 4 days post shift no small GFP-positive cells remained, and large weakly GFP-positive cells were observed (Fig. 3B). At 14 days post shift no GFP-positive cells remained in the midgut (Fig. 3C), with the exception of the gastric region stem cells (data not shown). Since *esg* expression is lost in differentiating EEs and ECs, it remained possible that *hnt* overexpression did not result in ISC loss through EC differentiation, but that the observed ISC loss is the result of ISC/EB delamination or death. To address this possibility, we repeated *hnt* overexpression experiments using the *esg^F/O^* technique to facilitate ISC lineage tracing (see Materials and Methods). Using *esg^F/O^* to express *UAS-GFP-hnt*, most GFP-positive cells appeared as large differentiated ECs that were integrated into the midgut epithelium at 3 days post shift. The lack of co-localization of Pros and GFP also confirmed that *hnt*-overexpressing cells do not differentiate as EEs (Fig. 3D). Control *esg^F/O^* midguts at 5 days post shift were typically found to contain clusters of GFP-positive cells consisting of several large cells as well as one or two small cells (Fig. 3E). At the same time point (5 days post shift), *hnt* overexpression using *hnt^EP55^* resulted in *esg^F/O^* lineages of only one or two cells that were Pdm1-positive, consistent with EC differentiation (Fig. 3F). Overall, these results show that increased *hnt* expression can force ISC-to-EC differentiation.

### The overexpression of *hnt* as ‘differentiation therapy’ in the fly

**Fig. 4.**
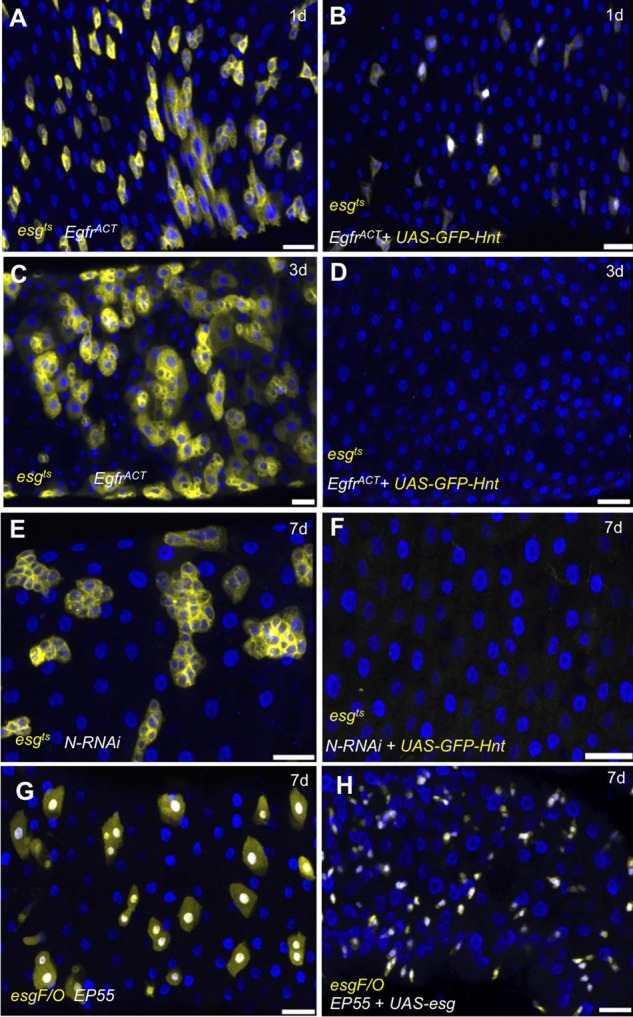
**Increased levels of Hnt can force EC differentiation in the context of ISC/EB overproliferation associated with reduced Notch signaling, midgut hyperplasia associated with activated Egfr signaling, but not in the context of increased Escargot.** (A-D) Anti-Hnt (blue) immunostained adult midguts expressing *UAS-GFP* (yellow) and *Egfr^ACT^* (A,C) or *Egfr^ACT^* and *GFP-Hnt* (B,D) are shown at 1 day (A,B) and 3 days (C,D) post shift (*esg^ts^* technique). *Egfr^ACT^* expression results in some hyperplasia 1 day post induction (A) and readily apparent hyperplasia 3 days post induction (C). *GFP-Hnt* co-expression abrogates all hyperplasia associated with activation of Egfr signaling (B,D). (E,F) Similar to above, anti-Hnt (blue) immunostained adult midguts expressing *UAS-GFP* (yellow) and *Notch-RNAi* (E) or *Notch-RNAi* and *GFP-Hnt* (F) are shown 7 days post shift. Reduced Notch signaling results in the overproliferation of small GFP-positive cells (E). GFP-Hnt co-expression abrogates overproliferation associated with reduced Notch signaling (F). (G,H) Anti-Hnt (blue) immunostained adult midguts expressing *UAS-GFP* (yellow) and *hnt^EP55^* (G) or *hnt^EP55^* and *escargot* (F) are shown 7 days post shift (*esg^F/O^* technique). ISC lineages overexpressing *hnt* typically consist of one or two large ECs (G, see also Fig. 3F). Co-expression of *escargot* and *hnt* results in no large cells. Scale bars: 20 µm.

The successful treatment of some forms of cancer involves ‘differentiation therapy’, whereby treatment aims to force malignant cells to resume normal differentiation (Warrell et al., 1991). The adult *Drosophila* posterior midgut has emerged as a model for mammalian midgut homeostasis and displays remarkable parallels in terms of dysregulation and hyperplasia (Casali and Batlle, 2009; Hartenstein et al., 2010; Wang and Hou, 2010). As previously shown in the literature and repeated in this study, reduced Notch signaling in the *Drosophila* midgut results in overproliferation of ISC-like cells (Micchelli and Perrimon, 2006; Ohlstein and Spradling, 2006, 2007). The activation of Egfr signaling is also known to result in overproliferation (Biteau and Jasper, 2011; Buchon et al., 2010; Jiang and Edgar, 2009). Similar to differentiation therapy, we tested the ability of *hnt* overexpression to suppress each overproliferation background. Using the *esg^ts^* technique, we found that expression of *Efgr^ACT^* resulted in discernable hyperplasia in the posterior midgut region within 24 h of induction (Fig. 4A). Such hyperplasia was not evident in midguts co-expressing *UAS-GFP-hnt* (Fig. 4B). By 3 days post induction *Egfr^ACT^* expression resulted in extensive hyperplasia (Fig. 4C) that was completely suppressed by *UAS-GFP-hnt* co-expression (Fig. 4D). Similarly, the overproliferation of ISC-like cells associated with the expression of *Notch-RNAi* (Fig. 4E) was suppressed by *UAS-GFP-hnt* co-expression at 7 days post induction (Fig. 4F). Overall, these results suggest that increased *hnt* expression can abrogate overproliferation by forcing ISCs to differentiate as ECs.

Recent results have established that *esg* is required for ISC maintenance (Korzelius et al., 2014), and this prompted us to determine if co-expression of *esg* could suppress the forced differentiation associated with *hnt* overexpression. Using the *esg^F/O^* technique, and similar to results shown in Fig. 3D (but here shown 7 days post-induction), *hnt^EP55^* overexpression resulted in the terminal differentiation of all ISCs as ECs, evident as large single or doublet GFP-positive cells (Fig. 4G). Co-expression of *UAS-esg* suppressed this effect, and no large, GFP-positive cells were observed (Fig. 4H). In summary, these results demonstrate that *hnt* overexpression is capable of promoting EC differentiation in a number of different contexts. The ability of *esg* overexpression to suppress EC differentiation, however, suggests that *hnt* overexpression alone is not sufficient for EC differentiation.

### *hnt* is required for EC differentiation in the adult midgut

**Fig. 5.**
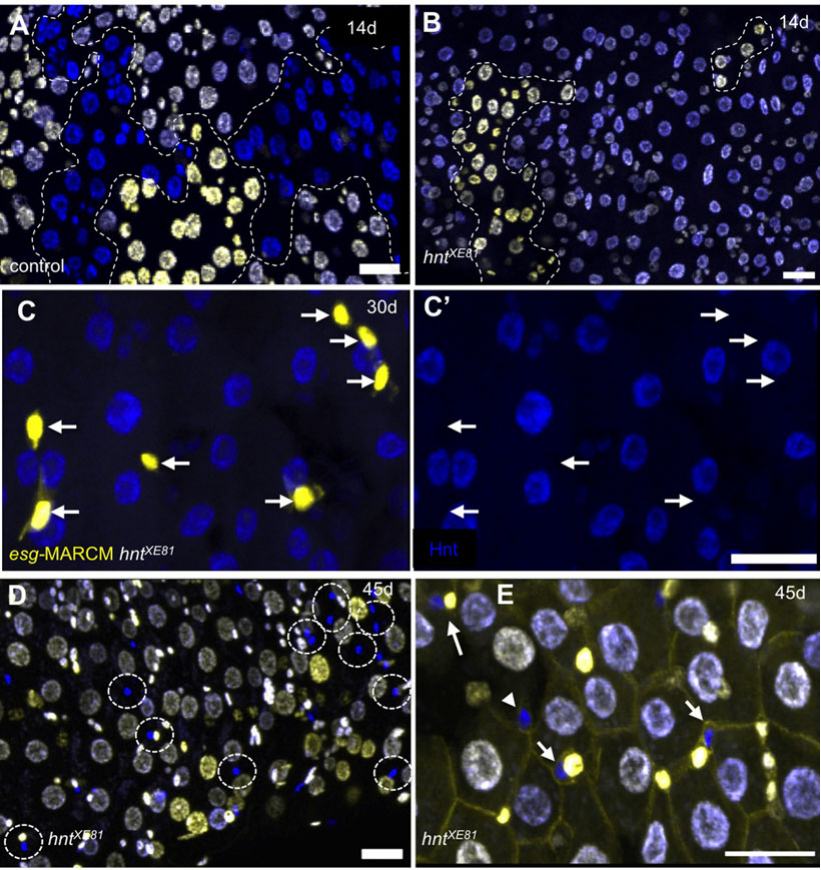
***hnt* is required for EC differentiation in the adult midgut.** (A,B) Twin spot analysis (see Materials and Methods) showing clones 14 days post induction in control (A) and *hnt^XE81^* (B). Clones are recognized by different levels of *His2Av-GFP* expression (yellow) where all cells express *His2Av-RFP* (blue) and are outlined by dashed lines. Control twin spots show both possible genotypes (yellow and blue), whereas clones generated in *hnt^XE81^* heterozygotes propagate only cells of the *hnt^+^* (yellow) genotype. (C,D) Anti-Hnt immunostaining in which *hnt^XE81^* mutant ISCs/EBs (*esg*-MARCM technique; see Materials and Methods) showing *hnt^XE81^* mutant ISCs/EBs 30 days post clone induction. GFP-positive cells (arrows in C) are Hnt-negative (arrows in C′). (D) In the context of the aged midgut, twin spot analysis reveals numerous small *hnt^XE81^* mutant cells (blue) 45 days post clone induction (dashed circles) and large *hnt^XE81^* mutant cells are not observed. (E) Anti-Arm immunostaining of twin spots showing *hnt^XE81^* mutant cells (blue) within the midgut epithelium apparent as either ISCs or EBs (arrows) or EEs (arrowhead). Scale bars: 20 µm.

**Fig. 6.**
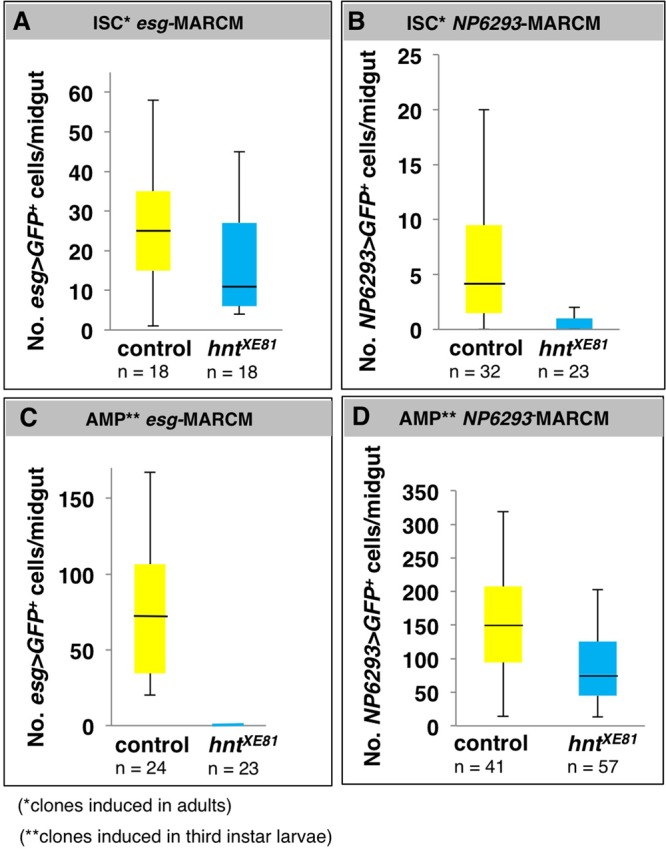
**Quantitation of *hnt^XE81^* loss of function marked clones induced in AMPs or ISCs using ISC/EB (*esgGAL4*) and EC (*NP6293*) specific reporters.** (A-D) Box plot diagrams are shown displaying the distribution GFP-positive cell counts using *esg*-MARCM (A,C) or *NP6293*-MARCM (B,D) to indicate adult ISC/EB and EC fates, respectively. The propensity of *hnt^XE81^* mutant cells to be recovered as ISCs/EBs following clone induction in ISCs (adult stage) is not markedly different from the control (A), whereas the propensity of *hnt^XE81^* mutant cells to differentiate as ECs following clone induction in adult ISCs is greatly reduced (B). Following clone induction in AMPs (during third instar larval stage) *hnt^XE81^* mutant cells are never recovered as ISCs/EBs in the adult midgut (C) whereas the same conditions result in ample *hnt^XE81^* mutant ECs (D). The median value is shown by a horizontal black line within each box. The median value for the recovery of *hnt^XE81^* mutant *NP6293*-MARCM positive cells following ISC clone induction (B) is zero (14/23 midguts with no GFP-positive cells). The median value for the recovery of *hnt^XE81^* mutant *esg*-MARCM positive cells following AMP clone induction (C) was also zero (23/23 midguts with no GFP-positive cells).

Having established that *hnt* overexpression can promote, but is not sufficient for EC differentiation, we wished to determine if *hnt* is necessary for this process. Our main approach for these experiments was to generate marked somatic clones within the ISC population of the adult midgut. Using a *FLP*/*FRT*-based twin spot technique, which permits analysis of all cell types, we first generated clones in which daughter cells either inherited two copies or no copy of an X-linked *His2Av-GFP* transgene marker in the background of a third chromosome carrying *His2Av-RFP* (see Materials and Methods). Following clone induction in mature adult females, twin spots composed of both small cells (presumptive ISCs/EBs or EEs) and large cells (presumptive ECs) were observed (Fig. 5A). Clones induced in *hnt^XE81^* heterozygotes, on the other hand, did not propagate the *hnt^XE81^* mutant side of the twin spot (RFP-only) and large *hnt^XE81^* mutant cells (presumptive ECs) were never observed (Fig. 5B). These observations suggested that *hnt* could be required for ISC maintenance and proliferation or EC differentiation. To distinguish between these two possibilities, a quantitative analysis was performed using a modified MARCM technique. Unlike the above GFP/RFP twin spot analysis, where small *hnt* mutant cells (RFP-only) were difficult to score, mutant cells in our MARCM-based mosaic analysis were readily observed. This technique (see Material and methods) generates GFP^nls^-labeled *hnt* mutant clones using either *esgGAL4* (*esg*-MARCM) or *NP6293* (*NP6293*-MARCM). This approach allowed us to evaluate the *hnt* mutant clones with respect to their ability to express either ISC/EB or EC specific markers. While the *esgGAL4* driver is well established as an effective reporter of ISCs/EBs, we have found *NP6293* to be an excellent reporter of EC fate throughout the adult midgut (Fig. S3). Using *esg*-MARCM and clone induction by heat shocking adults produced ample *esg>GFP*-positive cells throughout the midguts of both control and *hnt^XE81^* heterozygotes (Fig. 5C, Fig. 6A). Using *NP6293*-MARCM under identical conditions resulted in *NP6293>GFP*-positive cells in control, but not in *hnt^XE81^* heterozygotes (Fig. 6B). These results suggest that *hnt* is not required for ISC/EB maintenance and proliferation, but that there is a requirement for *hnt* in EC differentiation in the homeostatic adult midgut. Interestingly, we found that *hnt* mutant cells in the GFP/RFP twin spot analysis were more readily observed in the midguts of older flies (Fig. 5D). Anti-Arm immunostaining confirmed that these *hnt^XE81^* mutant cells are indeed part of the midgut epithelium. Based on morphology, most *hnt^XE81^* mutant cells resemble either ISCs or EBs, while some appear to be EEs (Fig. 5E). These observations further support our interpretation that *hnt* is not required in ISC maintenance or proliferation. In addition, even in the context of the aged midgut, which is prone to hyperplasia (Biteau et al., 2008; Choi et al., 2008), *hnt* mutant cells are incapable of EC differentiation.

Additional quantitative analysis compared *hnt* mutant ISC clones of the null allele *hnt^XE81^* to the hypomorphic allele *hnt^308^*. In this experiment, induced clones were marked by the expression of *UAS-GFP^nls^* under the control of both *esgGAL4* and *NP6293* (see Materials and Methods). Using this technique we measured the percentage of large GFP-positive cells, presumably indicative of differentiated ECs, at various time points after clone induction. Overall, large cells (EC-like) were most frequent in controls, significantly less frequent in *hnt^308^* clones, and were negligible in *hnt^XE81^* clones (Fig. S2A-C,E). Interestingly, while *hnt^308^* mutant clones contained a small percentage of large GFP-positive cells, anti-Pdm1 immunostaining showed these cells to be Pdm1-negative, suggesting that these large cells are not fully differentiated ECs (Fig. S2D).

Finally, to rule out the possibility that *hnt* ISC mutant clones result in increased EE differentiation, we measured the average number of Pros-positive cells in midguts harboring control, *hnt^XE81^*, and *hnt^308^* mutant ISC clones. We found no significant difference in the frequency of Pros-positive cells among the three genotypes, suggesting that *hnt* mutant ISC clones do not result in increased EE differentiation (Fig. S2F).

Taken together, both qualitative and quantitative analysis of somatic clones using two different *hnt* loss-of-function alleles allows us to conclude that differentiated ECs are not found within *hnt* mutant clones induced in ISCs. Therefore, *hnt* function is necessary for EC differentiation from the ISC/EB state in the adult midgut.

### *hnt* is not required for EC differentiation from the AMP state but is required for the establishment of ISCs from AMPs

**Fig. 7.**
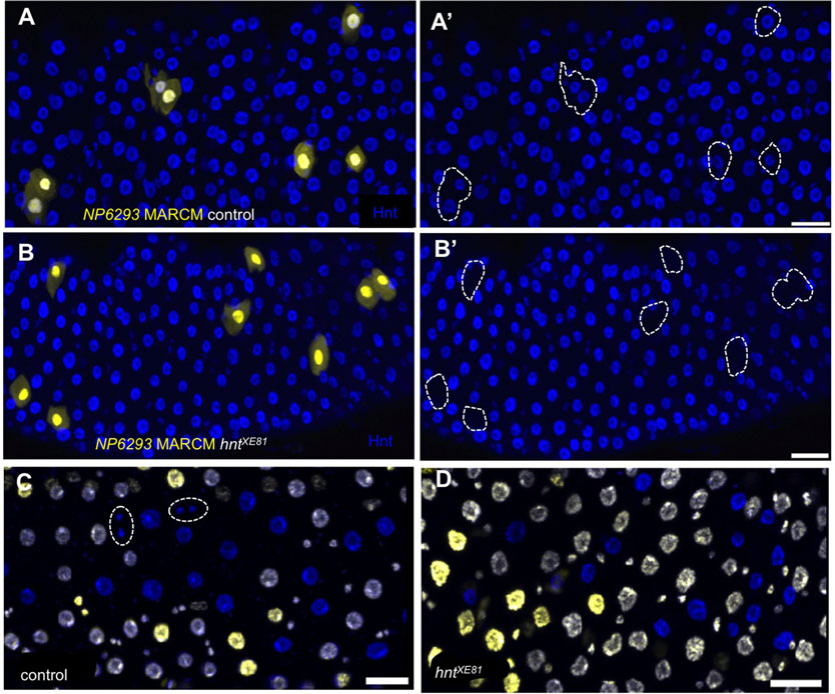
***hnt* is not required for AMP to EC differentiation but is required for the establishment of ISCs from AMPs.** (A) Anti-Hnt immunostaining of control midgut of 2-3 day old adult showing ECs marked by *NP6293*>*GFP^nls^* expression (*NP6293*-MARCM technique; see Materials and Methods) following clone induction during the third instar larval stage. Numerous large GFP-positive (yellow) and Hnt-positive (blue; dashed outline in A′) are found throughout the midgut. (B) Anti-Hnt immunostaining showing GFP-positive, Hnt-negative cells induced in *hnt^XE81^* heterozygotes (dashed outline in B′). (C,D) Twin spots induced in AMPs (larval heat shock) in control and *hnt^XE81^* showing numerous large mutant cells (blue) in both the control (C) and *hnt^XE81^* (D). Small mutant cells (blue) are evident in the control (C, dashed lines) but not in *hnt^XE81^*. Scale bars: 20 µm.

Having established that *hnt* is required for EC differentiation from the ISC/EB state, we wished to determine if *hnt* is generally required for EC differentiation, or if this requirement is specific to the adult ISC/EB. To address this question we repeated clonal analysis experiments in which clone induction was performed on third instar larvae. In so doing, *hnt* loss-of-function clones were induced among the population of proliferating AMPs. Most AMPs differentiate directly to ECs in the formation of the adult midgut, without passing through the ISC/EB state (Jiang and Edgar, 2009; Micchelli et al., 2011). Using *NP6293*-MARCM and clone induction by heat shocking third instar larvae produced ample *NP6293>GFP*-positive cells in both control and *hnt^XE81^* heterozygous (Fig. 7A,B; Fig. 6D). Using *esg*-MARCM under identical conditions resulted in numerous *esg>GFP*-positive cells in control, but not a single *esg>GFP*-positive cell was observed in *hnt^XE81^* heterozygous midguts (Fig. 6C). AMP clone induction by larval heat shock was also repeated using the GFP/RFP twin spot method as described above. Following clone induction in third instar larvae, twin spots composed of both small cells (presumptive ISCs/EBs or EEs) and large cells (presumptive ECs) were observed in the control (Fig. 7C). Clones induced in *hnt^XE81^* heterozygotes, on the other hand, did produce large EC-like *hnt^XE81^* mutant cells (RFP-only) but were devoid of small *hnt^XE81^* mutant cells (Fig. 7D). Overall, the ability of *hnt* mutant AMPs to differentiate into ECs suggests that *hnt* is not generally required for EC differentiation, but that *hnt* is required during the specific differentiation of ECs from the ISC/EB state in the adult midgut. In addition, the complete absence of *hnt* mutant ISCs in adults following clonal induction in AMPs strongly suggests that *hnt* is required for the establishment of viable or functional ISCs from AMPs.

## DISCUSSION

### *hnt* expression and Notch signaling

Our results demonstrate that *hnt* expression is maintained in ISCs depleted of Notch. While this establishes that *hnt* expression in ISCs is Notch-independent, it does not rule out the possibility that EC differentiation could require Notch-dependent *hnt* expression. For example, it remains possible that Notch signaling from the ISC to the EB could augment *hnt* expression in the EB, thereby promoting EC fate. Consistent with this possibility, our anti-Hnt immunostaining results suggest that *hnt* expression is increased in ECs relative to ISCs. In addition, our finding that *hnt* is not expressed in mature EEs also suggests that *hnt* is down-regulated as EBs enter the EE differentiation pathway, possibly reflecting reduced Notch signaling associated with EE differentiation. Taken together, our results do not rule out the possibility that *hnt* expression is Notch-dependent during ISC-to-EC differentiation.

### Hnt can promote but is not sufficient for ISC-to-EC differentiation

We report that co-expression of *hnt* and *esg* suppresses ISC-to-EC differentiation (compare Fig. 4G with H). Combined expression of *Notch^intra^* and *esg* was reported to partially suppress ISC-to-EC differentiation associated with the over-activation of Notch signaling (Korzelius et al., 2014). Our co-expression experiment, however, used *hnt^EP55^,* which expresses at a lower level than *UAS-GFP-hnt*. This difference in the level of expression was apparent in our *esg^ts^* experiments where *hnt^EP55^* was less effective than *UAS-GFP-hnt* in promoting ISC-to-EC differentiation (data not shown). It remains possible that co-expression of *esg* and a higher level of *hnt* overexpression could force ISC-to-EC differentiation, and that there is a threshold effect with respect to the level of Hnt and EC differentiation. The question as to whether Hnt and Esg compete for target genes, or if expression of either of these genes affects the regulation of the other remains unexplored at this time.

The JAK/STAT signaling pathway has also been implicated in the specification of EC cell fate (Beebe et al., 2010). Although not a main focus of our study, we found that RNAi knockdown of Stat92E had no effect on *hnt* expression. In addition, we found that co-expression of *UAS-GFP-hnt* with *UAS-Stat92E-RNAi* resulted in robust EC differentiation, suggesting that Hnt functions either in parallel or downstream of JAK/STAT signaling in EC differentiation (data not shown).

Overall, our analysis of *hnt* mutant clones supports the interpretation that *hnt* is necessary for ISC-to-EC differentiation. A recent genome-wide RNAi-based screen reported that depletion of Hnt using the *esg^ts^* technique results in ISC-to-EC differentiation (Zeng et al., 2015). This study, however, did not confirm EC differentiation using a reliable EC marker such as anti-Pdm1. Possible explanations for the discrepancy between our observations of *hnt* loss-of-function clones and the reported RNAi knockdown phenotype include RNAi off-site targets and possible differentiation defects. In the latter case, we suggest that the context of reduced Hnt associated with hypomorphic expression, such as we report in this study for *hnt^308^*, or incomplete RNAi penetrance, could result in premature dysregulation and misdifferentiation. Misdifferentiation and ISC overproliferation are associated with an activation of JNK signaling (Biteau et al., 2008). Interestingly, a proposed function of Hnt, albeit in a different context, is the down-regulation of JNK signaling (Reed et al., 2001).

### Hnt expression in ISCs is Egfr-dependent

One of the more surprising results in this study was that ISCs depleted of Egfr have greatly reduced Hnt, suggesting that *hnt* expression in this context is Egfr-dependent. In the scenario whereby *hnt* expression is positively regulated by Egfr signaling, we might expect similarities between *Egfr* and *hnt* mutant phenotypes. In support of this, we suggest that the phenotype of *hnt* mutant clones resembles *Egfr* mutant clones with respect to ISC proliferation and survival, and that these are dramatically different from the effects of reduced Notch signaling (Biteau and Jasper, 2011). The behavior of *hnt* and *Egfr* clones generated in the adult ISCs, however, differed with respect to cell differentiation. In mosaic analysis, and when using the MARCM technique, somatic recombination and subsequent mitotic division generate single cells that can be either mutant or wild type, presumably with equal probability. In the case of *Egfr* mutant clones, many newly formed single-cell clones can result in differentiated ECs or EEs, indicating that *Egfr* is not required for subsequent differentiation in the cases where the EB daughter cell is mutant (Biteau and Jasper, 2011). By contrast, *hnt^XE81^* mutant ISC clones generated in the adult midgut fail to differentiate as ECs. We have established that Hnt is required for ISC-to-EC differentiation, and the above clonal analysis suggests that *Egfr* is not required for EC differentiation. From this, we speculate that any expression or upregulation of *hnt* required to promote EC differentiation is most likely independent of Egfr signaling. This interpretation further supports the notion of a separate induction of *hnt* expression that is independent of Egfr signaling.

### Hnt is not required for AMP-to-EC differentiation but is required in the establishment of ISCs from AMPs

We show that Hnt is essential for ISC-to-EC differentiation, but not AMP-to-EC differentiation. As mentioned previously, Broad is known to play an important role in promoting AMP-to-EC differentiation, acting in parallel to the Notch signaling pathway. Additionally, Notch and Broad can effectively compensate for the loss of one another in allowing AMPs to differentiate as ECs (Zeng and Hou, 2012). We speculate that such AMP-to-EC differentiation in *hnt* mutant AMPs is occurring through the ecdysone/Broad pathway and this possibility merits further investigation.

Interestingly, we find that *hnt* mutant clones induced in AMPs never result in small, ISC or EE-like cells. This is unlike either the *broad* or *Notch* mutants. *broad* mutant clones induced in AMPs generate non-functional ISCs, which are Delta-positive but fail to proliferate or differentiate (Zeng and Hou, 2012). *Notch* mutant AMP clones, on the other hand, differentiate as EEs (Zeng and Hou, 2012). The absence of ISC or EE-like cells in *hnt* mutant clones suggests that *hnt* may play a primary role in the establishment of the ISCs from the AMP state. It also remains possible, however, that *hnt* mutant AMPs are preferentially eliminated by programmed cell death specific to the period of the larval-to-pupal transition. Interestingly, recent work in ovarian follicle cell differentiation has suggested that Broad functions together with Hnt in regulating the Notch-dependent mitosis-to-endocycle transition and cell differentiation (Jia et al., 2014). Given that *broad* mutant AMPs fail to generate fully functional ISCs (Zeng and Hou, 2012), and the complete lack of ISCs in *hnt* mutant AMP clones, allows us to speculate that, like the follicle cell context, Hnt and Broad may function cooperatively to establish the ISCs of the adult midgut.

## MATERIALS AND METHODS

### *Drosophila* stocks

All cultures were raised on standard *Drosophila* medium at 25°C under a 12 h light/dark cycle, unless otherwise indicated. Unless otherwise stated, stocks were obtained from the Bloomington *Drosophila* Resource Center. Controls were performed using *y w^1118^* or *y w^1118^ P{ry[+t7.2]=neoFRT}19A* stocks. *P{ry[+t7.2]=neoFRT}19A* is here abbreviated as *FRT19A*. The reporter line *w^1118^; P{w[+mC]=UAS-GFP.nls}14*, abbreviated in this report as *UAS-GFP^nls^*, was used to characterize *GAL4* expression patterns. The nuclear markers *His2Av-GFP* and *His2Av-RFP* are fully described as *P{w[+mC]=His2Av-EGFP.C}2* and *P{w[+mC]=His2Av-mRFP1}II.2* or *P{w[+mC]=His2Av-mRFP1}III.1*, respectively. The *UAS-Notch-RNAi* line used was *P{w[+mC]=UAS-N.dsRNA.P}14E*. The *UAS-EGFR-RNAi* line used was *w^1118^;; P{GD1654}v43267* and was obtained from the Vienna *Drosophila* RNAi Center. Most *escargot GAL4* lines used (*esgGAL4* only, with *UAS-GFP*, and with both *UAS-GFP* and *tubGAL80^ts^*) have been described previously (Micchelli and Perrimon, 2006). The *esg^F/O^* stock, described below, was provided by H. Jiang. The *hnt^308^*, *hnt^EP55^*, and *UAS-GFP-hnt* lines have been described (Ming et al., 2013; Reed et al., 2001). A recombinant *y w^1118^ hnt^XE81^ FRT19A* line was recovered in our lab. *UAS-Egfr^ACT^* was originally described as *UAS-λtop4.2* (Queenan et al., 1997) and was obtained from T. Schüpbach. The *NP6293 GAL4* line was obtained from the Kyoto *Drosophila* Resource Center. The *Yet1* enhancer trap line is from A. Michelson (Mohseni et al., 2009). The *UAS-esg* line from S. Hayashi was provided by J. Korzelius (Korzelius et al., 2014). *UAS-mCherry-moesin* was provided by R. Jacobs (McMaster University, Canada). Stocks used for mosaic analysis included *tubGAL80 hsFLP FRT19A* (full description: *P{w[+mC]=tubP-GAL80}LL1, P{ry[+t7.2]=hsFLP}1, P{ry[+t7.2]=neoFRT}19A*) as well as *Dp(1;2)4FRDup* originally from H. Salz (Case Western Reserve University, Ohio, USA).

### The *esg^ts^* technique

A method for inducing gene expression within the ISCs/EBs of the adult midgut has been described (Micchelli and Perrimon, 2006) and is here abbreviated as the *esg^ts^* technique. Briefly, this method uses a chromosome that carries the *esgGAL4* driver in addition to a *UAS-GFP* reporter and a *tubGAL80^ts^* insertion. Rearing cultures at 18°C, which is permissive for GAL80^ts^, prevents GAL4 activation and circumvents any effects associated with inducing gene expression during earlier stages of development. Shifting cultures to 29°C inactivates the GAL80^ts^ repressor, resulting in GAL4 activity and consequently *UAS-*reporter gene expression. To induce *UAS*-reporter gene expression in the ISCs, cultures were kept at 18°C and shifted to 29°C when adults were 3-5 days old. In all experiments midguts of females were analyzed. For co-expression *esg^ts^* experiments, virgin females carrying the X-linked *UAS-N-RNAi* or *UAS-Egfr^ACT^* insertions were crossed to males of the autosomal *UAS-GFP-hnt* stock. The male progeny of these crosses were subsequently crossed to *esg^ts^* virgin females.

### The *esg^F/O^* technique

ISC lineages were marked using the *esg^F/O^* (flip-out) technique (Jiang et al., 2009). Briefly, the *esg^F/O^* stock carries the same elements as the *esg^ts^* stock (*esgGAL4, UAS-GFP*, and *tubGAL80^ts^*) in addition to *UAS-Flp* (Flp recombinase) and a ubiquitous actin or tubulin based promoter designed to drive *GAL4* expression but prevented from doing so by a CD2 cassette flanked by FRT sites (i.e. *Act>CD2>GAL4*). Shifting cultures from 18°C to 29°C is permissive to *esgGAL4* expression of *UAS-Flp,* resulting the removal or ‘flip-out’ of the CD2 cassette. This permanently activates *ActGAL4* or *tubGAL4* within ISCs/EBs and their daughter cells. For *hnt* +* esg* co-expression *esg^F/O^* experiments, virgin females carrying the X-linked *hnt^EP55^* insertion were crossed to the autosomal *UAS-esg* line and resulting male progeny were crossed to *esg^F/O^* virgin females.

### Mosaic analysis: *His2Av-GFP*/*His2Av-RFP* twin spots

Experiments involving *His2Av-GFP*/*His2Av-RFP* twin spots used Bloomington stock 30563, fully described as *y w P{w[+mC]=His2Av[T:Avic\GFP-S65T]}1 P{ry[+t7.2]=hsFLP}122 P{ry[+t7.2]=neoFRT}19A; P{w[+mC]=His2Av-mRFP1}III.1/TM6B, Tb*. For the generation of control and *hnt^XE81^* mutant clones, males of the above stock were crossed to virgin females of *y w hnt^XE81^ FRT19A; Dp(1;2)4FRDup/+*. *Tb^+^* female progeny of this cross that carry *Dp(1;2)4FRDup*, which includes wild-type copies of both the *white* (*w*) and *hnt* genes*,* were used as controls. Sibling progeny lacking the duplication were used to generate *hnt^XE81^* mutant clones. For adult ISC clone induction, 3-5 day old adult female progeny were heat shocked twice for 40 min in a 37°C water bath, separated by a 1 h interval at room temperature. For larval AMP clone induction, progeny were heat shocked as described above when cultures contained wandering third instar larvae. For analysis of clones induced during larval stages (AMP clones), progeny eclosing either 5 or 6 days following heat shock treatment were dissected 1-2 days post-eclosion (cultures were maintained at 25°C following heat shock).

### Mosaic analysis: *esg*-MARCM and *NP6293*-MARCM techniques

Somatic clones were recovered using modified MARCM techniques (Lee and Luo, 2001). Rather than using a ubiquitous *GAL4* driver, *esgGAL4* and *NP6293 GAL4* drivers were used to positively mark clones as either ISCs/EBs or ECs, respectively. For *esg*-MARCM experiments *tubGAL80^ts^ hs-FLP FRT19A*; *esgGAL4 UAS-GFP^nls^*/*CyO* males were crossed to either virgin females of the *y w hnt^XE81^ FRT19A; Dp(1;2)4FRDup/+* or *y w hnt^XE81^ FRT19A/FM7* and *y w FRT19A* stocks. For *NP6293*-MARCM quantitative experiments *tubGAL80^ts^ hs-FLP FRT19A*; *NP6293 UAS-GFP^nls^ His2Av-RFP*/*CyO* males were crossed to the same *y w hnt^XE81^ FRT19A; Dp(1;2)4FRDup/+* (ISC *esg*-MARCM and AMP *NP6293*-MARCM) or *y w hnt^XE81^ FRT19A/FM7* and *y w FRT19A* stocks (ISC *NP6293*-MARCM and AMP *esg*-MARCM). For anti-Hnt *NP6293*-MARCM immunostaining experiment *tubGAL80^ts^ hs-FLP FRT19A*; *NP6293 UAS-GFP^nls^*/*CyO* females were crossed to *y w hnt^XE81^ FRT19A; Dp(1;2)4FRDup/+* males*.* All *esg*-MARCM and *NP6293*-MARCM experiments used the heat shock induction regimes as described above for *His2Av-GFP*/ *His2Av-RFP* twin spots, with the exception that adult heat shock treatment was repeated for 3-5 consecutive days and midguts were dissected 7 days following the final heat shock treatment.

Experiments comparing the behavior of *hnt^XE81^* and *hnt^308^* clones (Fig. S2) combined both *esgGAL4* and *NP6293-*MARCM techniques. Here GFP-positive clones indicate either ISCs/EBs due to *esgGAL4* expression (small GFP-positive cells) or ECs due to *NP6293* expression (large GFP-positive cells). Cells were scored as large if the nuclear diameter was greater than approximately 7 μm. The recovery of adult females carrying both *esg*- and *NP6293*-MARCM was achieved by crossing *y w hnt^XE81^ (or hnt^308^)*

*FRT19A; Dp(1;2)4FRDup/+* females to *NP6293 UAS-GFP^nls^ His2Av-RFP/ CyO* males to recover *y w hnt^XE81^ (or hnt^308^) FRT19A/ Y; Dp(1;2)4FRDup/ NP6293 UAS-GFP^nls^ His2Av-RFP* males. These males were subsequently crossed to *tubGAL80 hsFLP FRT19A*; *esgGAL4 UAS-GFP^nls^* females. Clones were induced as described for the GFP/RFP twin spot analysis. Following heat shock, flies were maintained at 25°C and transferred onto fresh food every 2-3 days. Adult females of the desired genotype (*y w hnt^XE81^ FRT19A/tubGAL80 hsFLP FRT19A; His2Av-RFP NP6293 UAS-GFP^nls^*/ *esgGAL4 UAS-GFP^nls^*) were identified by the absence of the *Confluens* phenotype associated with *Dp(1;2)4FRDup* in addition to using *His2AV-RFP* as a marker post-dissection. Females were dissected and midguts examined by live imaging confocal microscopy on days 3, 7, and 14 after clone induction. Control clones were similarly recovered by crossing *y w FRT19A*/*Y*; *His2Av-RFP NP6293 UAS-GFP^nls^*/+ males to *tubGAL80 hsFLP FRT19A*; *esgGAL4 UAS-GFP^nls^* females.

### Immunostaining

Immunostaining of adult midguts was carried out as described (Singh et al., 2012). The following primary antibodies were used at the indicated dilutions: mouse monoclonal anti-Hindsight (Hnt) 27B8 1G9 (1:25; from H. Lipshitz, University of Toronto, Canada); mouse monoclonal anti-Prospero (Pros) MR1A (1:100; DSHB); rabbit anti-Pdm1 (1:1000; from X. Yang, Zhejiang University, China); mouse monoclonal anti-Armadillo (Arm) N2 7A1 (1:100; DSHB). TRITC-conjugated goat anti-mouse and TRITC-conjugated donkey anti-rabbit secondary antibodies (1:500; Jackson Immunoresearch).

### Microscopy

For experiments that did not require immunolabeling, midguts were dissected in a drop of halocarbon oil (1:1 mixture of series 56 and series 700, Halocarbon Products Corp.), covered with a coverslip and imaged live. Immunostained midguts were mounted in 70% glycerol in PBS containing 2.5% DABCO (Sigma-Aldrich). Confocal microscopy and image processing were performed as previously described (Cormier et al., 2012).

### Quantitative analysis

To quantitate the *esg*-MARCM and *NP6293*-MARCM results shown in Fig. 6 GFP-positive cells were counted for the entire length of each midgut. Box plot diagrams were configured using Microsoft Excel, with whiskers indicating the minimum and maximum values of the data set. To quantitate the percentage of large GFP-positive cells shown in Fig. S2, the large GFP-positive cells and total GFP-positive cells were counted along the top surface for the entire length of each midgut. To quantitate EE frequency in midguts of control and midguts containing *hnt^308^* or *hnt^XE81^* mutant clones (shown in Fig. S2F) Pros-positive midgut cells within one field of view (20× objective) of the midgut/hindgut junction were counted. A minimum of five midguts was scored for each genotype. Standard deviation and *P* values were calculated using Microsoft Excel.
